# Molecular evolution and phylogenomic analysis of complete chloroplast genomes of *Cotinus* (Anacardiaceae)

**DOI:** 10.1002/ece3.10134

**Published:** 2023-05-29

**Authors:** Qiaoyun Liu, Nan Yang, Wenpan Dong, Liangcheng Zhao

**Affiliations:** ^1^ School of Ecology and Nature Conservation Beijing Forestry University Beijing China; ^2^ Museum of Beijing Forestry University Beijing Forestry University Beijing China

**Keywords:** adaptation, chloroplast genome, *Cotinus*, molecular evolution, phylogenetic relationship

## Abstract

*Cotinus* is an oligo‐specific ornamentally valuable genus with a disjunct distribution in the Northern Hemisphere. Traditionally, the taxonomy of *Cotinus* was mainly based on leaf morphological characteristics. However, the limited availability of genomic information greatly hindered the study of molecular evolution and phylogeny of this genus. This study sequenced the chloroplast (cp) genomes of all currently recognized taxa of *Cotinus*, including three species and four varieties. A comparative analysis was performed to investigate their cp genome characteristics and evolution. Furthermore, we inferred the phylogenetic relationships of *Cotinus* based on whole cp genomes, protein‐coding genes, and nuclear ITS data. All cp genomes exhibited a typical quadripartite structure with genome sizes ranging from 158,865 to 160,155 bp. A total of 113–114 genes were identified in the genomes. Seven non‐coding and four coding regions were identified as the most divergent hotspots for potential molecular barcodes and phylogenetic markers. Selection pressure analysis showed that there had been positive selection on genes *matK* and *rps8* in the *Cotinus* cp genomes. Phylogenetic results confirmed that *Cotinus* is a monophyletic group but the widely distributed species *Cotinus coggygria* is not monophyletic. The divergence‐time analysis suggested that *Cotinus* underwent an evolutionary divergence from the middle Eocene and rapid adaptive radiation from the middle Miocene. This study revealed new insights into the cp genome evolution and phylogeny of *Cotinus* and related taxa.

## Graphical abstract

Analyzed the plastid genome evolution of *Cotinus*, and identified 11 divergence hotspots for useful molecular barcodes. Conducted the selection pressure analysis for 79 protein‐coding genes. *Cotinus* is a monophyletic group but *Cotinus coggygria* is not monophyletic. *Cotinus* underwent a rapid adaptive radiation from the middle Miocene.

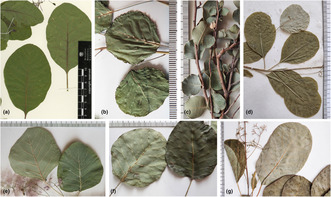

## 1 INTRODUCTION

*Cotinus* Mill. is an oligo‐specific genus in the family Anacardiaceae with great ornamental value. It exhibits a disjunct distribution in the Northern Hemisphere particularly in southern Europe, eastern Asia, and North America (Matić et al., 2016; Pell et al., 2011). The common name for *Cotinus*, “smoketree or smokebush”, refers to its unique large panicles, covered with tiny, persistent feathery plumes that give the inflorescences a wispy smoke‐like appearance. In addition, owing to their fast growth and drought tolerance, *Cotinus* species play a very important role in preventing soil erosion and soil water loss and contribute to the forest restoration and barren mountain afforestation in arid areas (Gavinet et al., 2019; Ge, 2014).

*Cotinus* was originally placed in the genus *Rhus* L. (Linneaus, 1753: 267), and later together with *Rhus*, *Actinocheita* Barkley, *Malosma* Nutt. ex Abrams, *Melanococca* Blume, *Metonium* Browne, *Searsia* Barkley, and *Toxicodendron* (Tourn.) Mill. were referred to as the *Rhus* complex (Barkley, 1963). The *Cotinus* taxonomy has always been determined mainly by its life‐form and leaf morphological characteristics (Cheng & Min, 1980; Li & Li, 2009; Min & Barfod, 2008; Penzes, 1958). The number of *Cotinus* species is debatable (Ferrer‐Gallego, 2016; Matić et al., 2016; Min & Barfod, 2008; Pell et al., 2011; Penzes, 1958), and generally four species were recognized, namely *Cotinus coggygria* Scop., *Cotinus obovatus* Raf., *Cotinus szechuanensis* A. Penzes, and *Cotinus nana* W. W. Smith (Pell et al., 2011).

**FIGURE 1:**
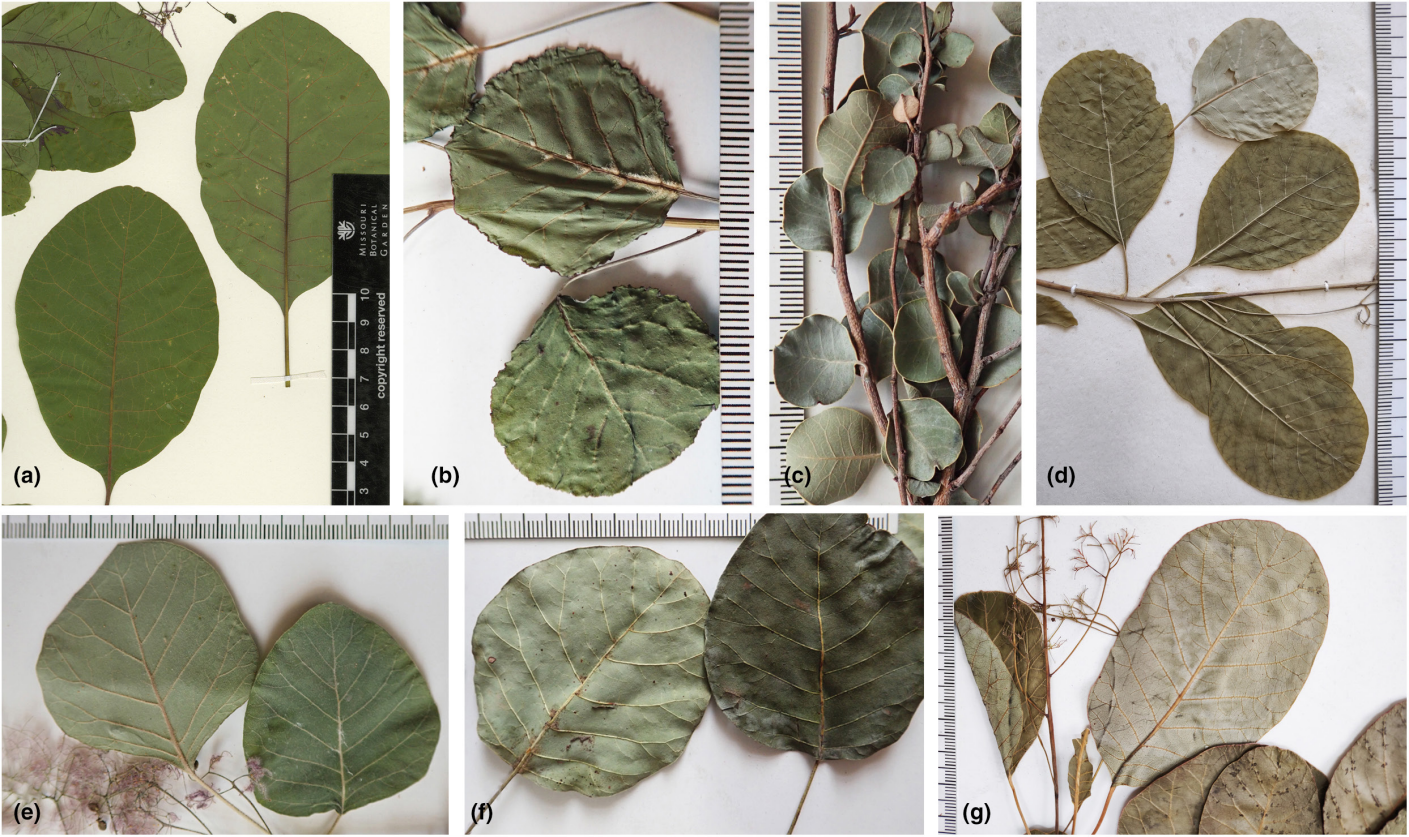
Leaf morphological characteristics of *Cotinus*. (a) *Cotinus obovatus*; (b) *Cotinus szechuanensis*; (c) *Cotinus nana*; (d) *Cotinus coggygria* var. *coggygria*; (e) *Cotinus coggygria* var. *cinerea*; (f) *Cotinus coggygria* var. *pubescens*; (g) *Cotinus coggygria* var. *glaucophylla*. (a) photographed from the specimen (No. 6406192) in Missouri Botanical Garden Herbarium (MO); (d) photographed from the specimen (No. 00021667) in Beijing Forestry University Herbarium (BJFC); others photographed from the field materials collected by Liangcheng Zhao.

*Cotinus coggygria* is the most economically important and widespread species in nature and cultivation. This plant is well‐known for its ornamental horticulture value in both inflorescences and leaves and for being an important source of essential oils and extracts with medicinal properties (Matić et al., 2016). It is distributed from southern Europe into the Mediterranean region and, with discontinuities, across the continent through the Himalayas and into China (Gavinet et al., 2019; Miao et al., 2017). *Cotinus coggygria* is a large shrub or small tree with a variable habitat and diverse leaf morphology. The leaves are 3–8 × 3–6 cm in size, broadly elliptic, suborbicular, obovate, or ovate in outline, glabrous or pubescent. Due to the great variability of leaf morphological characteristics, *C*. *coggygria* was generally further divided into four varieties: *C*. *coggygria* var. *coggygria*, *C*. *coggygria* var. *cinerea* Engl., *C*. *coggygria* var. *pubescens* Engl., and *C*. *coggygria* var. *glaucophylla* C. Y. Wu (Cheng & Min, 1980; Min & Barfod, 2008). The four varieties are more or less different in leaf shape and pubescence (Min & Barfod, 2008; also see Figure 1). However, whether these varieties are established or monophyletic needs further study. *Cotinus obovatus* is restricted to the southeastern United States and naturally grows on rocky, calcareous soils in a few hilly and mountainous areas. Its leaves are obovate in outline (Figure 1a) and can be 13 cm in length, generally larger and coarser than those of *C*. *coggygria* (Hoagland, 2004). The other two species, *C*. *szechuanensis* and *C*. *nana*, are both restricted to the narrow areas of Southwest China. *Cotinus szechuanensis* is characterized by its relatively small leaves, 2–6 × 2–5 cm in size, almost round in outline with a wavy margin, glabrous adaxially but abaxially with conspicuous tufts of hair in vein axils (Figure 1b). Its native habitat in the northwestern Sichuan province is usually a small shrub in dry, high‐elevation mountainous regions (Li et al., 2007). *Cotinus nana* is found only in the dry and open areas of hill thickets and grasslands in the southwestern Yunnan province (Min, 1979). This species is a low, compact shrub with a height less than 1 m. Its leaves are leathery and small (0.5–2 cm in diameter), almost round in outline, glabrous both adaxially and abaxially (Figure 1c).

The delimitation of species by leaf morphology has been controversial because these traits are easily affected by the environment, and convergence and parallel evolution often occur (Singh, 2010). Therefore, using phenotypic traits alone to infer phylogenetic relationships between taxa with different genotypes is problematic, especially within such an inherently variable genus. Although molecular sequence data have been widely used as phylogenetic tools for decades, there are few molecular studies devoted to establishing relationships in *Cotinus*.

Using more molecular data is essential to infer the phylogenetic relationships of *Cotinus*, especially from a genomic perspective. Chloroplast (cp) genomes are circular DNA molecules composed of a large single copy (LSC) region and a small single copy (SSC) region separated by a pair of inverted repeats (IR) regions (Sugiura, 1992). Cp genome is independent of the nuclear genome and has highly conserved gene content, number, and structure (Daniell et al., 2016). Over the past years, it has been used to elucidate plant molecular evolution, enhancing our understanding of chloroplast biology, conservation, and diversity (Daniell et al., 2016). Moreover, cp genomes represent rich information sources for phylogenomics, from higher‐level to below the species level (Barrett, 2020; Dong et al., 2021, 2022; Ruhfel et al., 2014). To date, the cp genome is available for only one representative *Cotinus* species. Xu et al. (2021) reported the complete cp genome of *C*. *coggygria* and conducted a comparative analysis of some other genera in Anacardiaceae.

Here, we sequenced and assembled the cp genomes of all currently recognized species and varieties in *Cotinus*. A comparative analysis of the complete cp genomes was conducted to quantify the cp genomic variation among the species of *Cotinus*. Furthermore, a phylogenomic analysis was performed based on whole cp genomes to infer the phylogenetic position of *Cotinus* in Anacardiaceae and the phylogeny among the species. In addition, the nuclear ITS sequences were used to construct the phylogeny to observe the phylogenetic congruence or incongruence between nuclear and cp genome data. Specifically, we attempted to (1) reveal the cp genome characteristics, and divergence patterns of *Cotinus*; (2) provide useful cp genomic resources for molecular barcodes and phylogenetically informative markers; and (3) improve our understanding of the evolutionary history and infrageneric relationships of *Cotinus*.

## 2 MATERIALS AND METHODS

### 2.1 Plant material, DNA extraction and genome assembly

Ten accessions representing three species and four varieties of *Cotinus* were sampled and sequenced. The fresh leaves of *C*. *nana*, *C*. *szechuanensis*, *C*. *coggygria* var. *cinerea*, *C*. *coggygria* var. *glaucophylla* and *C*. *coggygria* var. *pubescens* were collected from the field and dried with silica gel. Voucher specimens were deposited at the Beijing Forestry University Herbarium (BJFC), Beijing. The dry leaves of *C*. *coggygria* var. *coggygria* were sampled from the specimen in the Beijing Forestry University Herbarium. The dry leaves of *C*. *obovatus* was provided by Prof. Libing Zhang from Missouri Botanical Garden, USA. Details of the sampling information were shown in Table A1.

Total genomic DNA was extracted from silica‐dried leaves using a modified CTAB protocol (Doyle & Doyle, 1987) and purified employing the Wizard DNA Clean‐Up System. DNA samples were fragmented randomly and then were sheared into 350 bp fragments through agarose gel electrophoresis. The bp insert size paired‐end libraries with 350 were built using the Illumina PE DNA library kit, and then paired reads were sequenced with an Illumina HiSeq 4000.

The cp genome assembly were performed using GetOrganelle v1.6.2d (Jin et al., 2020). The GetOrganelle first filtered plastid‐like reads, conducted the de novo assembly, purified the assembly, and finally generated the complete cp genomes (Bankevich et al., 2012; Camacho et al., 2009; Langmead & Salzberg, 2012). K‐mer gradients for a mean and maximum 150 bp reads were set to “‐k 21, 45, 65, 85,105” for all taxa. Bandage (Wick et al., 2015) was used to visualize the final assembly graphs to authenticate the automatically generated cp genome. Gene annotation was conducted using Plann (Huang & Cronk, 2015) with the annotated cp genome of *Pistacia chinensis* (NC_046786.1) as the reference genome. These cp genome sequences were deposited in GenBank (ON167899–ON167909; Table A1). The nuclear ITS sequences were assembled from the clean data of same samples using GetOrganelle as the cp genome assembly.

### 2.2 Comparative genomic analyses

Cp genome maps were drawn using Chlorobox (Tillich et al., 2017). Drawing the IR/SC boundaries regions map display the IR expansion and contraction among *Cotinus* cp genomes. The mVISTA program (http://genome.lbl.gov/vista/mvista/about.shtml; Frazer et al., 2004) was performed using the annotation of *C*. *coggygria* var. *cinerea* as the reference to analyze genomic structural variation. Visual results from mVISTA were further analyzed using the alignment model LAGAN, which producing true multiple alignments regardless of whether they contain inversions (Brudno et al., 2003).

### 2.3 Nucleotide divergence and selection pressure estimation

To screen polymorphic hotspots that can be used as molecular markers for species identification of *Cotinus*, 78 shared coding regions (CDS) and 103 intergenic spacer regions (IGS) of the seven cp genomes were separately extracted, aligned using MAFFT (Katoh et al., 2019), and manually adjusted using MEGA 7 (Kumar et al., 2016). Subsequently, the aligned sequence length, number of variable sites, and parsimony‐informative characters (PICs) for each region were calculated using DnaSP 5.0 (Librado & Rozas, 2009). We used two methods to identify mutation hotspots. The first was based on the percentage of variable sites, which calculated all the single‐nucleotide substitutions and included the most divergence markers. The second was based on the percentage of PICs, which included the most informative markers. To estimate selection pressures, non‐synonymous (Ka) and synonymous (Ks) substitution rate ratios (ω = Ka/Ks) of the 78 protein‐coding genes were calculated using the CodeMl program in the PAML v4.9 (Gao et al., 2019; Xu & Yang, 2013). We applied three site‐evolution models to test whether some sites (codons) are under positive selection: the M0 (one ratio), M7 (β) and M8 (β and ω). Likelihood ratio tests (LRTs) with *p*‐value < .05 were performed to test the statistical significance.

### 2.4 Phylogenetic analyses

To assess the phylogenetic relationships of *Cotinus*, we established three datasets and used Maximum likelihood (ML) and Bayesian Inference (BI) methods for phylogenetic analysis. The first dataset WCG included 25 whole cp genomes from Anacardiaceae and two outgroup genomes from Burseraceae (10 newly generated and 17 obtained from GenBank; Table A2). The second dataset PCG comprised 76 protein‐coding genes (excluding two genes under positive selection) in the cp genomes. The third ITS dataset included 24 nuclear ITS sequences (10 newly created with the cp genome and 14 obtained from GenBank; Table A2). All the sequences were aligned with MAFFT (Katoh et al., 2019), and ambiguous alignment regions were trimmed by Geneious 2021 with default parameters (Sites containing: Gaps = 20%; Ripma et al., 2014).

The best‐fitting nucleotide substitution model was determined using the ModelFinder (Kalyaanamoorthy et al., 2017). The ML trees were used RAxML v.8.2.12 (Stamatakis, 2014) software, and the reliability of the phylogenetic tree topology adopts 1000 self‐expanding times analysis and inspection (bootstrap support values, BS). The BI tree was generated using the MrBayes 3.2.6 software (Ronquist et al., 2012), based on Markov Chain Monte Carlo (MCMC) process. Four chains were run in parallel for 1,000,000 generations, sampling once every 1,000 generations, discarding the first 25% of the trees, and verifying them by Bayesian posterior probabilities (PP).

### 2.5 Dating divergence times

We estimated the divergence times using relaxed‐clock method implemented in the BEAST version 1.8.2 (Drummond et al., 2012) and the whole cp genomes dataset. Three fossils were used as a priori to calibrate the divergence time. Wood fossils related to Anacardiaceae and Burseraceae were reported from the upper Cretaceous of Mexico (Estrada‐Ruiz et al., 2010). We set the root age (the divergence time between Anacardiaceae and Burseraceae) to be 70 million years ago (Ma). This age was consistent with the hypothetical diversification time of Burseraceae (De‐Nova et al., 2012). The reliable fruit fossils of *Rhus* were found in the middle Eocene of western North America (Manchester, 1994). The minimum stem age of *Rhus* was calibrated to be 44 Ma. The oldest fossil records of *Pistacia* were found in the Oligocene of Europe and North America (Ramirez & Cevallos‐Ferriz, 2002), and this fossil was used to calibrate the minimum stem age of *Pistacia* to be 33 Ma. These three fossil priors were given lognormal prior distributions with an offset of the fossil ages, a mean of 1.5 and a standard deviation of 1, allowing for the possibility that these nodes are considerably older than the fossils themselves.

BEAUti v2.4.7 was used to create the input file for Beast. The GTR + G alternative model and the relaxed log‐normal clock model were set to run for a total of 40 million generations, sampling every 1,000 generations, ensuring that the parameters converged in Tracer v.1.7 (Rambaut et al., 2018) with valid sample size values >200. The first 20% of the resulting trees set were rounded off and the remainder were used to construct the Maximum Clade Credibility (MCC) tree in TreeAnnotator v.1.10.4.

## 3 RESULTS

### 3.1 Chloroplast genome characteristics of *Cotinus*

**FIGURE 2:**
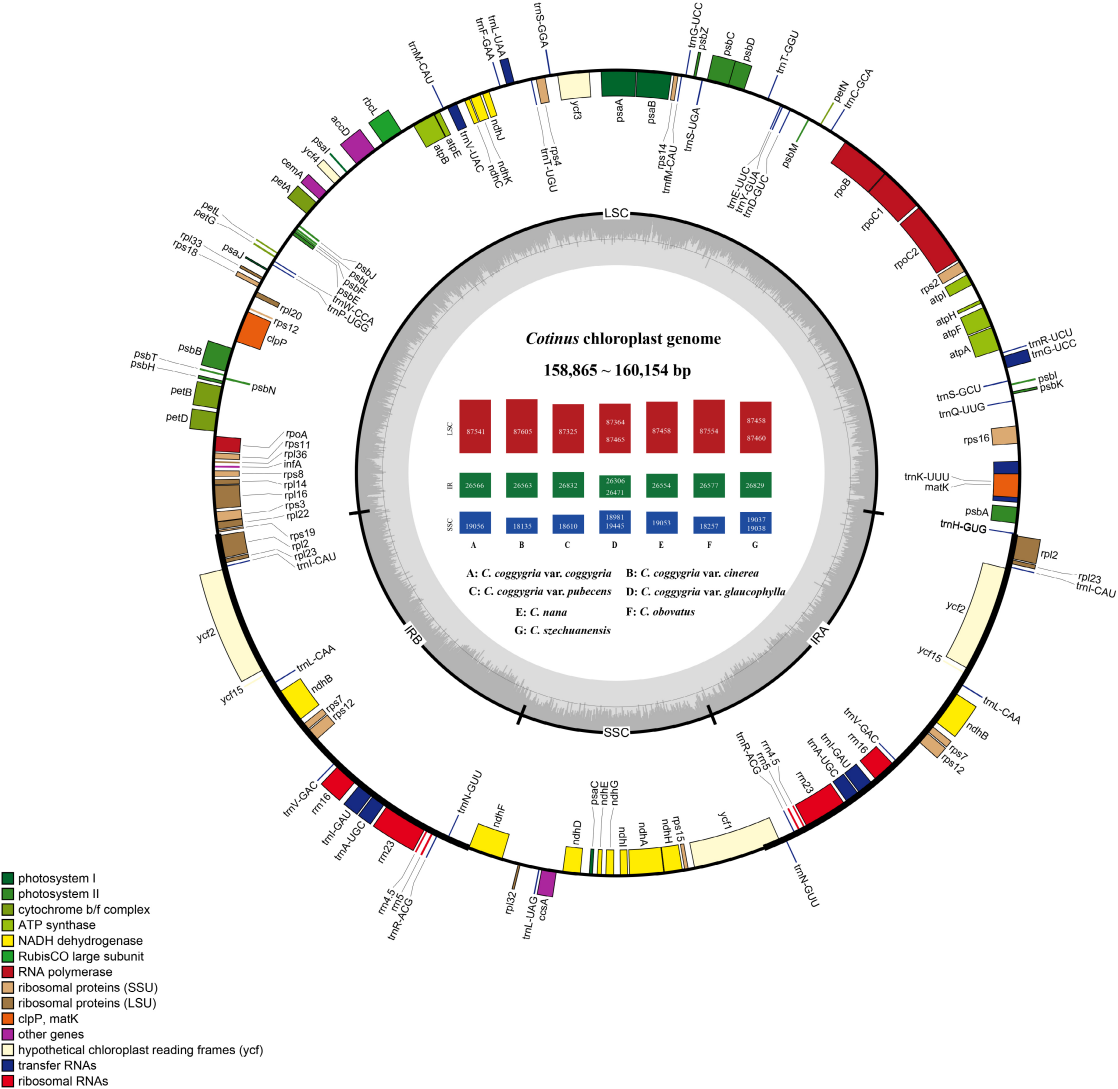
Gene map of *Cotinus* cp genomes. Genes shown inside the inner circle are transcribed counterclockwise and those outside are transcribed clockwise. Genes belonging to different functional groups are colour‐coded. Darker grey in the inner circle corresponds to the GC content of the cp genome.

We assembled and compared 10 whole cp genomes from three species and four varieties of *Cotinus*. All cp genomes were typical covalently closed, double‐stranded circular molecules that contained an LSC region of 87,325–87,605 bp, an SSC region of 18,135–19,445 bp, and a pair of IR regions of 26,306–26,832 bp (Figure 2 and Table 1). The cp genome sizes ranged from 158,865 bp in *C*. *coggygria* var. *glaucophylla* to 160,155 bp in *C*. *nana*. The total GC content was 37.9%–38.1%. There are 113–114 unique genes (including 78–80 protein‐coding genes). *Cotinus szechuanensis* and *C*. *coggygria* var. *glaucophylla* lost the *rpl32* gene, and the *rps19* gene was lost in *C*. *obovatus* and *C*. *coggygria* var. *pubescens* cp genomes. All the cp genomes had 30 tRNA genes and four rRNA genes. Seven protein‐coding genes (*rps19*, *rpl2*, *rpl23*, *ycf2*, *ycf15*, *ndhB*, and *rps7*) in five taxa (except *C*. *obovatus* and *C*. *coggygria* var. *pubescens*), seven tRNA genes (*trnI‐CAU*, *trnL‐CAA*, *trnV‐GAC*, *trnI‐GAU*, *trnA‐UGC*, *trnA‐UGC*, and *trnR‐ACG*) and four rRNA genes (*rrn16*, *rrn23*, *rrn4*.*5*, and *rrn5*) in all genomes were duplicated in the IR regions (Figure 2 and Table 2).

**TABLE 1:** Comparsion of 10 newly sequenced cp genomes of *Cotinus*.

	*Cotinus coggygria* var. *coggygria*	*Cotinus coggygria* var. *cinerea* 053	*Cotinus coggygria* var. *cinerea* 004	*Cotinus coggygria* var. *pubescens*	*Cotinus coggygria* var. *glaucophylla*	*Cotinus nana* 003	*Cotinus nana* 052	*Cotinus obovatus* 057	*Cotinus szechuanensis* 055	*Cotinus szechuanensis* 044
Genome size (bp)	159,729	159,287	159,422	159,599	158,865	160,154	160,155	159,619	158,965	158,965
LSC length (bp)	87,541	87,364	87,365	87,325	87,605	87,458	87,460	87,458	87,554	87,554
SSC length (bp)	19,056	18,981	19,445	18,610	18,135	19,038	19,037	19,053	18,257	18,257
IR length (bp)	26,566	26,471	26,306	26,832	26,563	26,829	26,829	26,554	26,577	26,577
Coding (bp)	90,561	90,682	90,912	90,834	90,078	90,606	90,564	90,271	90,364	90,364
Intron size (bp)	19,009	19,328	19,118	19,239	19,327	19,133	19,133	19,166	19,123	19,123
Spacer size (bp)	50,159	49,277	49,392	49,526	49,460	50,415	50,458	50,182	49,478	49,478
Number of genes	114	114	114	113	113	114	114	113	113	113
Number of protein‐coding genes	80	80	80	79	79	80	80	78	79	79
Number of tRNA genes	30	30	30	30	30	30	30	30	30	30
Number of rRNA genes	4	4	4	4	4	4	4	4	4	4
GC content (%)	38	38	38	37.9	38.1	37.9	37.9	37.9	38	38
LSC of GC (%)	36.1	36.1	36.1	36.1	36.2	36.1	36.1	36.1	36.1	36.1
SSC of GC (%)	32.5	32.6	32.6	32.6	32.8	32.5	32.5	32.5	32.8	32.8
IR of GC (%)	42.9	43	43.1	42.8	43	42.8	42.8	43	42.9	42.9

**TABLE 2:** Genes located in *Cotinus* cp genomes.

Category of genes	Group of genes	Gene name
Self replication	Ribosomal RNAs	rRNA16^c^, rRNA23^c^, rRNA4.5^c^, rRNA5^c^
	Transfer RNAs	trnA‐UGC^a^ ^,^ ^c^, trnC‐GCA, trnD‐GUC, trnE‐UUC, trnF‐GAA, trnfM‐CAU, trnG‐UCC^a^, trnH‐GUG^e^, trnI‐CAU^c^, trnI‐GAU^a^ ^,^ ^c^, trnK‐UUU^a^, trnL‐CAA^c^, trnL‐UAA^a^, trnL‐UAG, trnM‐CAU, trnN‐GUU^c^, trnP‐UGG, trnQ‐UUG, trnR‐ACG^c^, trnR‐UCU, trnS‐GCU, trnS‐GGA, trnS‐UGA, trnT‐GGU, trnT‐UGU, trnV‐GAC^c^, trnV‐UAC^a^, trnW‐CCA, trnY‐GUA
	Ribosomal protein (small subunit)	rps2, rps3, rps4, rps7^c^, rps8, rps11, rps12^a^ ^,^ ^d^, rps14, rps15^c^, rps16^a^, rps18, rps19^c^
	Ribosomal protein (large subunit)	rpl2^a^ ^,^ ^c^, rpl14, rpl16^a^, rpl20, rpl22, rpl23^c^, rpl32, rpl33, rpl36
	DNA dependent RNA polymerase	rpoA, rpoB, rpoC1, rpoC2
	Translation‐related gene	infA
Genes for photosynthesis	Subunits of photosystem	psaA, psaB, psaC, psaI, psaJ
	Subunits of photosystem	psbA, psbB, psbC, psbD, psbE, psbF, psbH, psbI, psbJ, psbK, psbL, psbM, psbN, psbT, psbZ
	Subunits of cytochrome b/f complex	petA, petB^a^, petDa, petG, petL, petN
	Subunits of ATP synthase	atpA, atpB, atpE, atpF^a^, atpH, atpI
	Subunits of NADH dehydrogenase	ndhA^a^, ndhB^a^ ^,^ ^c^, ndhC, ndhD, ndhE, ndhF, ndhG, ndhH^c^, ndhI, ndhJ, ndhK
	ATP‐dependent protease subunit	clpP
	Rubisco large subunit	rbcL
Other genes	Maturase	matK
	Subunit of acetyl‐CoA‐carboxylase	accD
	Envelop membrane protein	cemA
	c‐type cytochrome biogenesis	ccsA
	Conserved open reading frames	ycf1, ycf2^c^, ycf3^b^, ycf4, ycf15^c^

^a^ Gene containing a single intron.

^b^ Gene containing two introns.

^c^ Two gene copies in the IRs.

^d^ Gene divided into two independent transcription units.

^e^ Duplicated gene in LSC region.

### 3.2 IR contraction and expansion

**FIGURE 3:**
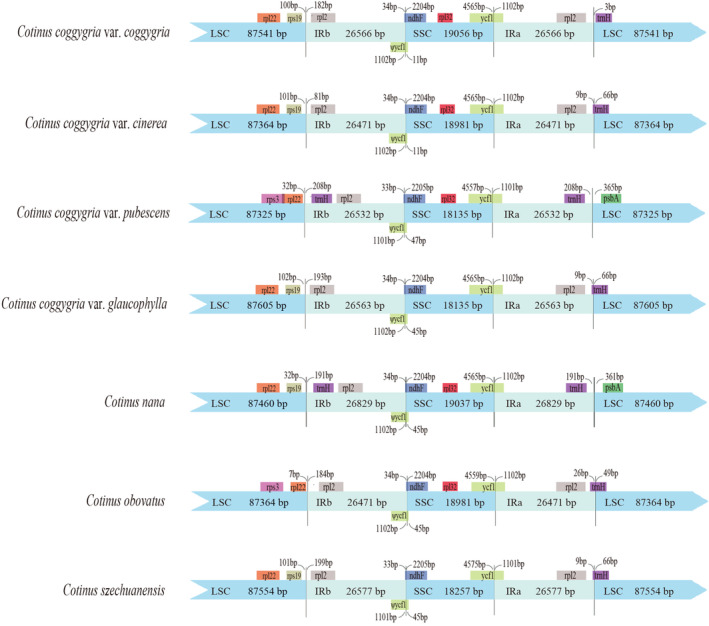
Comparison of the borders of large single copy (LSC), small single copy (SSC), and inverted repeats (IR) regions in cp genomes of seven *Cotinus* taxa. Different colour boxes indicate specific genes.

To elucidate the putative contraction and expansion of the IR regions in *Cotinus* cp genomes, we investigated the gene variation at the IR/SC boundary regions (Figure 3). In the LSC/IRb border, the *rpl2* gene in the LSC region was all expanded completely to the IRb region. In the IRb/SSC border, the *ndhF* gene all crossed the junctions and extended into the IRb region, with a length from 33 to 34 bp. In the SSC/IRa border, the junctions were all crossed by the *ycf1* gene with a length of 1101–1102 bp. In the IRa/LSC border, the *trnH* gene crossed the junctions to various degrees.

*Cotinus coggygria* var. *coggygria*, *C*. *coggygria* var. *cinerea*, *C*. *coggygria* var. *glaucophylla*, *C*. *szechuanensis*, and *C*. *obovatus* had identical SC/IR and boundary variations. The IRa/LSC junctions were located between *rpl2* and *trnH*, and LSC/IRb junctions between *rps19* and *rpl2*. However, *C*. *coggygria* var. *pubescens* and *C*. *nana* showed significant variations in LSC/IRb and SSC/IRa boundaries compared to the other five taxa. The *trnH* extend from the LSC to the IR region, resulting in the IRa/LSC junctions all located between *trnH* and *psbA*. The LSC/IRb junctions were located between *rpl22* and *trnH* in *C*. *coggygria* var. *pubescens*, and between *rps22* and *rpl2* in *C*. *obovatus*, because the *rps19* gene was lost in these two cp genomes.

### 3.3 Sequence divergence and mutation hotspot regions

**FIGURE 4:**
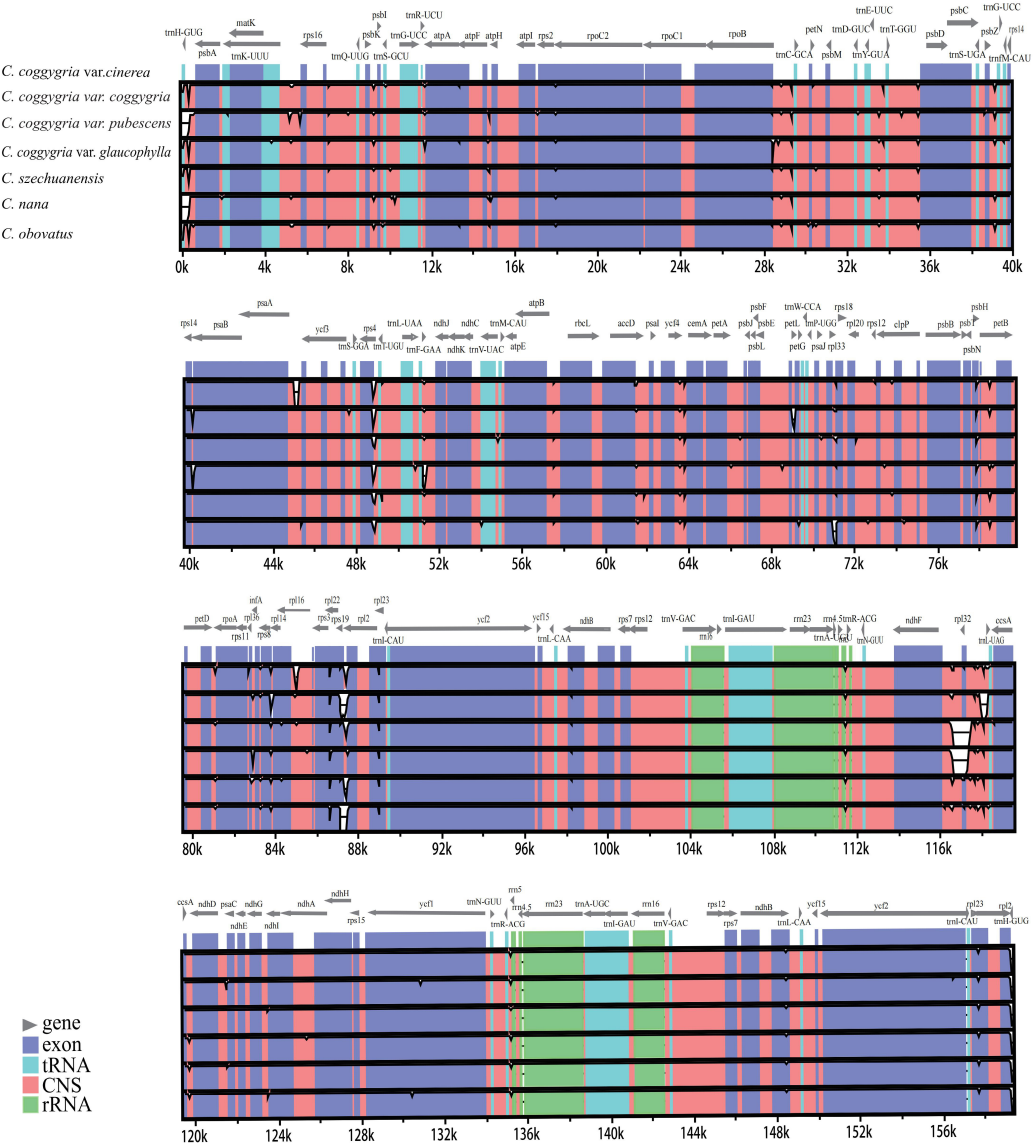
Sequence alignment of the seven *Cotinus* cp genomes using mVISTA under the LAGAN model.

To quantify sequence divergence, the whole cp genomes from the seven taxa of *Cotinus* were compared with mVISTA. The alignments revealed high sequence similarity across these cp genomes (Figure 4). The loss of *rpl32* gene led to the gaps in the alignments for *C*. *szechuanensis* and *C*. *coggygria* var. *glaucophylla*, and the loss of *rps19* gene led to the gaps for *C*. *obovatus* and *C*. *coggygria* var. *pubescens*. The average variable percentages of the whole genome, coding and non‐coding regions were 0.88%, 0.59%, and 1.14%, respectively, with the highest percentage in the SSC region (1.40%), followed by the LSC region (1.27%), and then the IR region (only 0.10%; Table 3). The PICs percentages of the whole genome, coding and non‐coding regions were 0.11%, 0.04%, and 0.21%, respectively. The SSC region had the highest PICs percentage (0.16%), followed by the LSC region (0.14%), and the IR region (only 0.01%; Table 3).

**TABLE 3:** Statistics for genomic divergence among the seven *Cotinus* cp genomes.

Gene regions	Type	Aligned length of sequences (bp)	Variable sites		Parsimony informative sites	
			Number	%	Number	%
Whole cp genome	–	161,875	1,424	0.88	174	0.11
All coding	–	91,254	534	0.59	34	0.04
All noncoding	–	71,584	816	1.14	151	0.21
SSC	–	19,174	268	1.40	30	0.16
LSC	–	88,959	1,130	1.27	125	0.14
IRs	–	26,920	28	0.10	3	0.01
*atp*A‐*atp*F	Non‐coding	57	6	10.53	4	7.02
*rps*16‐*trn*Q(UUG)	Non‐coding	1,567	160	10.21	2	0.13
*rpl*33‐*rps*18	Non‐coding	209	20	9.57	0	0
*rpl*36‐*inf*A	Non‐coding	222	18	8.11	1	0.45
*atp*F‐*atp*H	Non‐coding	289	22	7.61	12	4.15
*rps*8‐*rpl*14	Non‐coding	244	15	6.15	0	0
*trn*K(UUU)‐*rps*16	Non‐coding	1,107	64	5.78	7	0.63
*rps*18	Coding	306	32	10.45	1	0.33
*rpl*22	Coding	521	38	7.29	8	1.54
*rpl*32	Coding	175	8	4.57	1	0.57
*rpl*36	Coding	114	4	3.51	0	0

**FIGURE 5:**
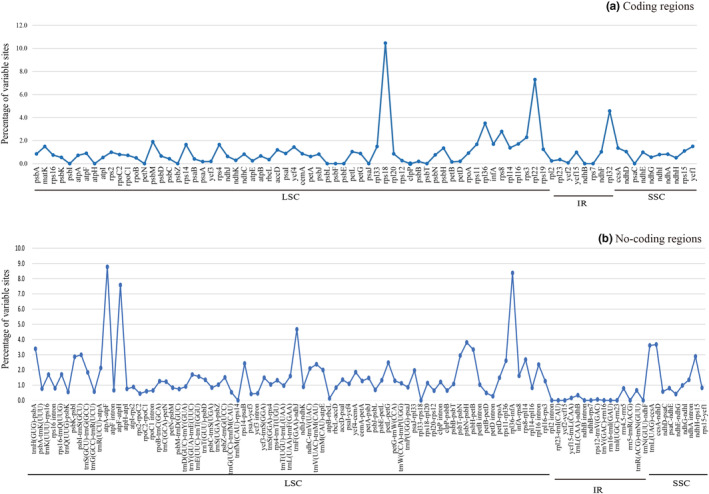
Percentage of variable sites in aligned sequences of the seven *Cotinus* cp genomes. (a) Coding region; (b) noncoding region.

To identify the mutation hotspot regions, the percentages of variable sites and PICs were calculated in each coding and non‐coding region (Figure 5 and Table 3). We defined a mutation hotspot region as having a percentage of variable sites >5% for non‐coding regions and >3% for coding regions. Seven non‐coding genes (*atpA‐atpF*, *rps16‐trnQ* ^*UUG*^, *rpl33‐rps18*, *rpl36‐infA*, *atpF‐atpH*, *rps8‐rpl14* and *trnK* ^*UUU*^ *‐rps16*) and four coding genes (*rps18*, *rpl22*, *rpl32* and *rpl36*) were identified as the most divergent hotspot regions. These hotspot regions were all located in the LSC region except for *rpl32* located in the SSC region. Concerning PICs, gene regions *atpA‐atpF* (7.02%) and *atpF‐atpH* (4.15%) had higher PICs percentages in non‐coding regions, while gene *rpl22* had a higher percentage (1.54%) in coding regions.

### 3.4 Selection pressure analysis

**FIGURE 6:**
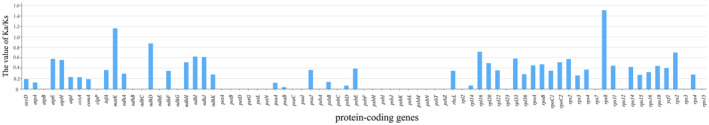
The non‐synonymous/synonymous substitution rates (Ka/Ks) calculated using 78 protein‐coding genes in seven *Coinus* cp genomes.

Using the site‐evolution models of CodeMl, non‐synonymous to synonymous nucleotide substitution rate ratios (Ka/Ks) for 78 shared protein‐coding genes were calculated (Figure 6). Among these genes, the Ka/Ks ratios had a range from 0 to 1.5070 with an average value of 0.2471. The Ka/Ks ratios showed eight genes (*atpE*, *ndhG*, *petG*, *psaI*, *psbH*, *psbK*, *ycf3*, and *ycf15*) with Ka/Ks = 0, and no gene with Ka/Ks = 1. Only two genes (*matK* and *rps8*) had Ka/Ks ratios greater than 1, with the values of 1.1605 and 1.5070, respectively. The remaining 68 genes showed Ka/Ks ratios of between 0.0001 and 0.8709.

### 3.5 Phylogenetic analysis

**FIGURE 7:**
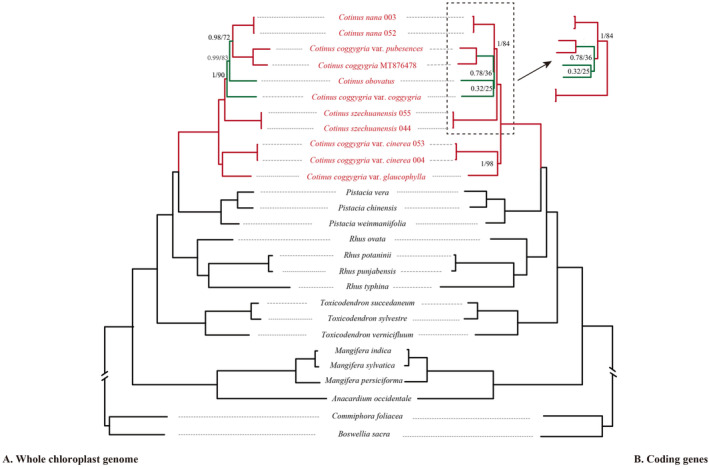
Phylogenetic reconstruction of *Cotinus* and related taxa using Bayesian inference (BI) and maximum likelihood (ML) methods based on WCG (a) and PCG (b), respectively. The green branches represent the different topology between WCG and PCG. The support values for the nodes are for posterior probabilities (PP) and bootstrap support (BS). Branches with no values listed have 100% bootstrap support.

The phylogenomic analyses were conducted based on WCG and PCG datasets (Figure 7). The WCG tree displayed higher supported posterior probability (PP) and bootstrap (BS) values at most nodes that provided better phylogenetic relationships of *Cotinus* and related genera. However, the supported values of some nodes within *Cotinus* were moderate or low, especially for the PCG tree.

Both WCG and PCG datasets showed that the sampled taxa of *Cotinus* clustered together, forming a monophyletic group, sister to *Pistacia* with high support (PP/BS: 1/100). Three species and four varieties formed two clades. One clade comprised *C*. *nana*, *C*. *coggygria* var. *pubescens*, *C*. *coggygria* (MT876478), *C*. *obovatus*, *C*. *coggygria* var. *coggygria* and *C*. *szechuanensis*. Within this clade, *C*. *coggygria* var. *pubescens* was sister to *C*. *coggygria* (MT876478) with high support (PP/BS: 1/100). In the WCG tree, *C*. *coggygria* var. *pubescens* and *C*. *coggygria* were sister to the two individuals of *C*. *nana* with moderate support (PP/BS: 0.98/72), then together sister to *C*. *obovatus* with moderate support (PP/BS: 0.99/83). This group was, in turn, sister to *C*. *coggygria* var. *coggygria* with higher support (PP/BS: 1/90). In the PCG tree, *C*. *coggygria* var. *coggygria* was sister to *C*. *obovatus* with low support (PP/BS: 0.32/25), which sister to the *C*. *coggygria* var. *pubescens‐C*. *coggygria* (MT876478) group with low support (PP/BS: 0.78/36), and then together sister to *C*. *nana* with higher support (PP/BS: 1/84). In both WCG and PCG trees, the two individuals of *C*. *szechuanensis* were the firstly diverged species in this clade with strong support. The second clade consisted of *C*. *coggygria* var. *glaucophylla* and two individuals of *C*. *coggygria* var. *cinerea*. There were high support values for the second clade in both WCG and PCG trees (PP/BS: 1/100 and PP/BS: 1/98, respectively).

**FIGURE 8:**
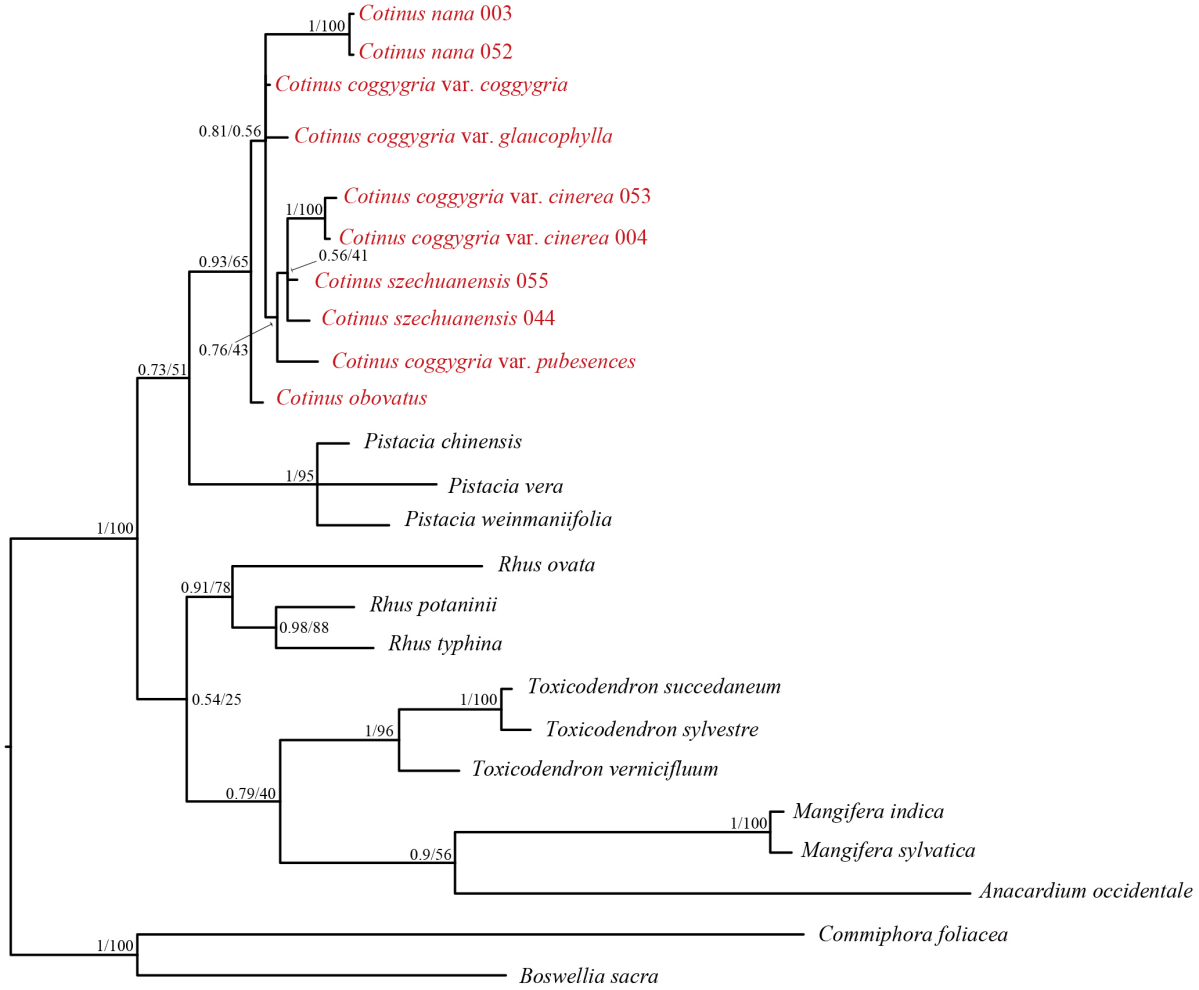
Phylogenetic reconstruction of *Cotinus* and related taxa using Bayesian inference (BI) and maximum likelihood (ML) methods based on ITS sequences. The support values for the nodes are for posterior probabilities (PP) and bootstrap support (BS). Branches with no values listed have 100% bootstrap support.

The ITS dataset also supported that all the taxa of *Cotinus* formed a monophyletic group but most nodes were lower supported within *Cotinus* (Figure 8). In addition, extensive incongruences between cp genome and nuclear data occurred in both species and varieties levels. For example, ITS data showed that *C*. *obovatus* was the firstly diverged group in *Cotinus*.

### 3.6 Divergence time dating

**FIGURE 9:**
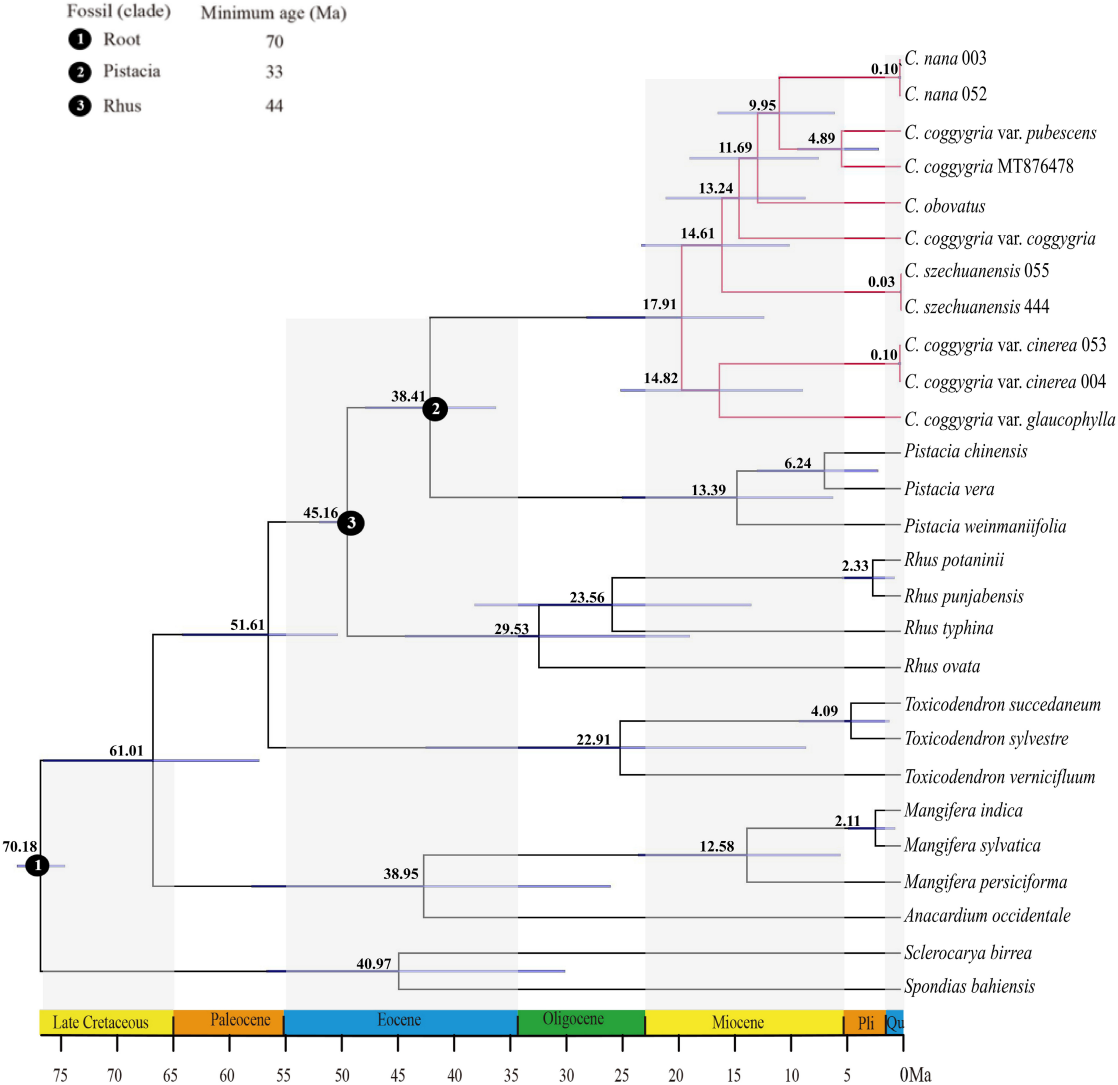
Divergence times of *Cotinus* and related taxa based on the whole cp genomes and fossil calibrations. Branches in bold indicate 95% HPD boundary. Circles 1–3 at the nodes represent fossil constraints.

Based on whole cp genomes data, the mean estimated ages and 95% HPD (highest posterior density) intervals of nodes were mapped onto the dating chronogram (Figure 9). The stem age of *Cotinus* was estimated to be 38.41 Ma (95% HPD: 33.04–43.69 Ma) during the middle Eocene, and the crown age of *Cotinus*, leading to the divergence of the genus into two clades, was estimated at 17.91 Ma (95% HPD: 11.29–25.66 Ma) in the middle Miocene. Within *Cotinus*, *C*. *coggygria* var. *glaucophylla* and *C*. *coggygria* var. *cinerea* were estimated to diverge at 14.82 Ma (95% HPD: 8.04–22.89 Ma) in the middle Miocene, and *C*. *szechuanensis* was suggested to diverge around similar time (14.61 Ma, 95% HPD: 9.12–21.18 Ma). *Cotinus coggygria* var. *coggygria* was estimated to diverge at 13.24 Ma (95% HPD: 7.82–19.20 Ma), and *C*. *obovatus* was estimated at 11.69 Ma (95% HPD: 6.74–17.24 Ma). *Cotinus nana* diverged from *C*. *coggygria* var. *pubescens* at 9.95 Ma (95% HPD: 5.41–14.97 Ma) in the late Miocene.

## 4 DISCUSSION

### 4.1 Chloroplast genome evolution of Cotinus

This study sequenced and compared 10 cp genomes representing all currently recognized taxa of *Cotinus* to elucidate cp genome evolution. All the genomes had a typical quadripartite structure, and there were no rearrangements in genome organization, highly similar to other Anacardiaceae chloroplast genomes. The cp genomes sizes from the seven *Cotinus* taxa varied from 158,865 to 160,155 bp. The total GC content was similar to related Anacardiaceae species, such as *Rhus chinensis* Mill. (37.79%), *Pistacia weinmannifolia* Poiss. ex Franch. (37.84%), and *Toxicodendron vernicifluum* (Stokes) F. A. Barkley (37.96%; Wang et al., 2020; Zheng et al., 2017). The average GC contents of the IR regions were higher than in the LSC and SSC regions, indicating that rRNA and tRNA, found mainly in IR regions, prefer to use G and C bases (Li et al., 2016).

The contraction and expansion of IR generally lead to gene order change in the cp genomes of angiosperms and play important roles in genome evolution (Wicke et al., 2011). The inclusion of three species and four varieties in *Cotinus* showed that their IR boundaries were variable at and below the species levels. Large IR expansions in these cp genomes caused the *rpl2* gene to transform to the IRb region completely, and the *trnH* gene transformed to the IRa region to various degrees. IR expansion also led to the partial duplication of *ycf1* in the IRb region, thereby generating a *ycf1* pseudogene located in the IRb/SSC border.

Gene loss often occurs during the evolution of angiosperms cp genomes (Daniell et al., 2016; Jansen et al., 2008). It has been proposed that contraction/expansion of the IR regions could lead to gene loss/addition (Wicke et al., 2011). Among the examined cp genomes, the *rpl32* gene was lost in *C*. *szechuanensis* and *C*. *coggygria* var. *glaucophylla*, and the *rps19* gene was missing in *C*. *obovatus* and *C*. *coggygria* var. *pubescens*, indicating that gene deletion occurred during cp evolution. The missing *rps19* gene from the cp genomes was also reported in some species in *Rhus* (Barrett, 2020). Some lost genes were proved to be transferred to the nuclear or mitochondrial genomes, where they may still be expressed (Daniell et al., 2016). For example, the *rpl32* gene transfers to the nucleus in *Populus* and *Thalictrum* (Park et al., 2015; Ueda et al., 2007). It is unknown whether the loss of *rps19* and *rpl32* from the cp genomes of *Cotinus* species is due to gene transfer to the nucleus/mitochondrion or loss from the cell entirely.

Mutation hotspots can provide valuable variable sequence information for species identification and phylogeny research (Dong et al., 2021, 2022). Most sequence mutation hotspots occurred in the non‐coding regions across the cp genomes of the seven *Cotinus* taxa, indicating the coding regions were more conserved than the non‐coding ones, consistent with most angiosperm cp genomes (Daniell et al., 2016). We identified seven non‐coding and four coding regions as the most divergent hotspots in *Cotinus*. All hotspots are located in the LSC and SSC regions and the IR regions showed higher conservation than the LSC and SSC regions. These divergent regions may be undergoing rapid nucleotide substitution, indicating great potential molecular markers for phylogeny and evolutionary research in *Cotinus*.

Mutations accompany species adaptations to diverse environments under positive selection (Dong et al., 2018; Yin et al., 2018). The Ka/Ks ratio in provides important insights into adaptive molecular evolution, and reveals positive selection signals in plant cp genes (Dos Reis, 2015; Hermida‐Carrera et al., 2017). This ratio represents the selective pressures acting on the protein‐coding gene with values of ω = 1, ω < 1, and ω > 1, being indicative of neutral evolution, purifying selection and positive selection, respectively. In the 78 shared protein‐coding genes of seven *Cotinus* cp genomes, we found that the average Ka/Ks value was 0.2471, and 68 genes had Ka/Ks values less than 1, implying that at the cp genome level, most genes were subjected to purifying selection. Eight genes had Ka/Ks values of 0, suggesting they were under strong purifying selection to retain conserved functions. The genes of *matK* and *rps8* showed Ka/Ks values greater than 1, suggesting that the two genes may have undergone positive selective pressures. The *matK* gene is located within the *trnK* intron, and functionally may be involved in splicing group II introns coding for tRNALys (Crawley & Hilu, 2012). Due to the unknown function, *rps8* is a valuable resource for future research of the adaptive evolution of *Cotinus*.

### 4.2 Phylogeny of *Cotinus*

This study used WCG, PCG and ITS datasets to construct the phylogenetic relationships of *Cotinus* and related taxa. Sampling covered all currently recognized species and varieties of *Cotinus*, contributing to an understanding of its intrageneric relationships and evolution. Phylogenetic tree based on WCG had obviously higher support than that constructed from PCG because the whole genomes have more variable and informative sites than the coding regions. In the ITS tree, due to the inadequate variations, most of the support values were low, and the ITS data could not resolve the interspecific relationships within *Cotinus*.

The phylogenetic analyses based on all the datasets supported *Cotinus* as a monophyletic group distinct from *Rhus* and *Toxicodendron*, confirming the exclusion of *Cotinus* from the *Rhus* complex, as documented in previous studies (Jiang et al., 2019; Miller et al., 2001; Yi et al., 2004). Our analyses also supported the sister relationship of *Cotinus* and *Pistacia*, consistent with previous molecular studies (Jiang et al., 2019; Xie et al., 2014; Xu et al., 2021; Yang et al., 2016). The divergence time estimated using WCG dataset suggested that *Cotinus* diverged from *Pistacia* ca. 38.41 Ma, close to the diversification time of 37.6 Ma (Xie et al., 2014) and 37.11 Ma (Jiang et al., 2019) estimated using several nuclear and plastid markers. These results indicated that *Cotinus* originated during the middle Eocene.

Within *Cotinus*, due to its greatly diverse leaf characteristics, the widely distributed *C*. *coggygria* was generally divided into four varieties. The phylogenetic results showed that *C*. *coggygria* was not monophyletic because the four varieties did not form one clade, but were interspersed with other species. Our *C*. *coggygria* var. *pubescens* sample from Chongqing, southwestern China clustered with *C*. *coggygria* MT876478 [also sampled from Chongqing by Xu et al. (2021)] with significant support, suggesting that they most likely were the same variety. *Cotinus coggygria* var. *pubescens* had a closer relationship with *C*. *nana* than other varieties. *Cotinus coggygria* var. *coggygria* was at the base of *C*. *coggygria* var. *pubescens*‐*C*. *nana* and *C*. *obovatus* clade, with a divergence time ca. 11.69 Ma. *Cotinus coggygria* var. *cinerea* was sister to *C*. *coggygria* var. *glaucophylla*, forming the first divergent group of the entire genus, diverging with other taxa at ca. 17.91 Ma, indicating evolutionary radiation within *Cotinus* from the middle Miocene to present. A more definitive taxonomic treatment of these varieties requires further studies.

For the other three species, *C*. *nana* was sister to *C*. *coggygria* var. *pubescens*, but they have different leaf morphologies. *Cotinus nana* is characterized by its extremely compact habitat and very small leaves, remarkably different from other species and varieties. In geographic distribution, *C*. *nana* is restricted to a very narrow range of Jinsha River Basin in southwestern Yunnan Plateau, a mountain region with a dry‐hot valley climate (high temperatures and lack of available water; Zhang et al., 2002, 2013). Whether the exceptionally habitat and leaf size of this species are functions of the harsh environment is unknown. Very little is known about the ecological adaptation and evolution of *C*. *nana*, and further investigation is necessary. *Cotinus obovatus* was nested within the clade of *C*. *coggygria* var. *coggygria* and *C*. *coggygria* var. *pubescens*‐*C*. *nana*. Morphologically, *C*. *obovatus* is similar to *C*. *coggygria* var. *coggygria* in having glabrous leaves but different by its oblong‐obovate leaf shape with somewhat acuminate tip and larger leaf size. *Cotinus szechuanensis* is at the base of the {*C*. *coggygria* var. *coggygria*, [*C*. *obovatus*, (*C*. *coggygria* var. *pubescens*, *C*. *nana*)]} clade. *Cotinus szechuanensis* is similar to *C*. *coggygria* var. *pubescens* and *C*. *coggygria* var. *coggygria* in habitat and leaf shape but is distinguished by its smaller leaves with conspicuous tufts of white pubescence in the primary vein axils on their lower surface. Additionally, its leaves may have a wavy margin. In geographic distribution, *C*. *szechuanensis* is restricted to the high‐mountain (>2,000 m) regions of the arid river valley in northwestern Sichuan. Based on the analysis on leaf characteristics of *C*. *szechuanensis* and environmental factors in the dry valley of the upper Minjiang River, Li et al. (2007) suggested that drought and high temperature in the leaf growth period primarity affect the anatomical characteristics. *Cotinus szechuanensis* divergence time was estimated at ca. 14.61 Ma in the middle‐late Miocene, and it was probably geographically isolated from the *C*. *coggygria* var. *pubescens* and*/*or *C*. *coggygria* var. *coggygria* predecessors during this period in which the Himalaya‐Tibetan plateau was accelerated uplifted (Fang, 2017; Wen et al., 2014).

In our analyses, two individuals of *C*. *nana*, *C*. *szechuanensis* and *C*. *coggygria* var. *cinerea* from different localities clustered with extremely short terminal branches, indicating that they were highly homologous respectively. On the other hand, the phylogenetic trees had very short internode connected by long branches at the interspecific nodes, suggesting that *Cotinus* may have undergone rapid speciation and adaptive radiation during the evolution process. Phylogenetic reconstruction may be difficult due to hybridization/introgression, incomplete lineage sorting (ILS), and reticulation evolution. This is especially true for the groups in which speciation was accompanied by rapid adaptive radiation (Azarin et al., 2021; Meng et al., 2021; Rosenberg & Nordborg, 2002). In our WCG tree, the phylogenetic relationships among *C*. *coggygria* var. *coggygria*, *C*. *obovatus*, *C*. *coggygria* var. *pubescens* and *C*. *nana* were still not fully resolved due to moderate support values. Moreover, there was incongruence concerning the relationships of these four taxa between WCG, PCG and ITS data. This is probably due to their rapid radiation or reticulate evolution during the period of the late Miocene.

## 5 CONCLUSIONS

The present study investigated the characteristics and evolution of the cp genomes from three species and four varieties in *Cotinus*, representing all currently recognized taxa of this genus. Comparing these genomes revealed that the genome structure and gene content were fairly conserved among these taxa. However, the IR boundaries were variable at both species and varieties levels, which was related to IR expansion and gene loss. The sequence divergence and mutation hotspots of these cp genomes were analyzed. Seven non‐coding and four coding regions had the highest divergence and may be considered useful molecular barcodes and phylogenetic markers. Selection pressure analysis showed that there had been significant positive selection on *matK* and *rps8* genes in the cp genomes of *Cotinus*. The phylogenetic analysis based on whole cp genomes, protein‐coding genes and nuclear ITS data confirmed that the genus *Cotinus* is a monophyletic group but the widely distributed species *C*. *coggygria* is not monophyletic. In addition, the divergence‐time analysis revealed evolutionary radiation of *Cotinus* from the middle Miocene. This study revealed new insights into the cp genome evolution and phylogeny of *Cotinus* and related taxa.

## AUTHOR CONTRIBUTIONS

**Qiaoyun Liu:** Conceptualization (equal); data curation (lead); formal analysis (lead); investigation (equal); methodology (equal); resources (equal); software (equal); writing – original draft (equal). **Nan Yang:** Data curation (supporting); formal analysis (supporting); investigation (equal); methodology (equal); resources (equal). **Wenpan Dong:** Conceptualization (equal); formal analysis (equal); methodology (equal); software (equal); supervision (equal); writing – original draft (equal). **Liangcheng Zhao:** Conceptualization (equal); funding acquisition (lead); investigation (equal); project administration (lead); resources (equal); supervision (lead); validation (equal); writing – original draft (equal); writing – review and editing (lead).

## FUNDING INFORMATION

This research was supported by Science and Technology Basic Resources Investigation Program of China “Survey and Germplasm Conservation of Plant Species with Extremely Small Populations in South‐west China” (Grant No. 2017FY100100).

## CONFLICT OF INTEREST STATEMENT

The authors declare that there is no conflict of interest.

## Data Availability

The 10 newly sequenced chloroplast genomes were submitted to the NCBI database (https://www.ncbi.nlm.nih.gov/) with GenBank accession numbers ON167899–ON167909.

## APPENDIX 1

**TABLE A1:** Sample information of the 10 newly sequenced *Cotinus* cp genomes.

Species	Sample locality	Voucher (herbarium)	GenBank accession
*Cotinus coggygria* var. *coggygria*	Marche, Italy	ZZX3609	ON167909
*Cotinus coggygria* var. *cinerea*	Jiufeng, Beijing, China	BJFUZLC053	ON167901
*Cotinus coggygria* var. *cinerea*	Kunyushan, Shandong Province, China	BJFUZLC004	ON167902
*Cotinus coggygria* var. *pubescens*	Jinfoshan, Chongqing, China	BJFUZLC001	ON167905
*Cotinus coggygria* var. *glaucophylla*	Lijiang, Yunnan Province, China	BJFUZLC040	ON167903
*Cotinus nana*	Xianggelila, Yunnan Province, China	BJFUZLC003	ON167900
*Cotinus nana*	Xianggelila, Yunnan Province, China	BJFUZLC052	ON167899
*Cotinus obovatus*	Missouri Botanical Garden, USA	ZLB11126	ON167904
*Cotinus szechuanensis* 055	Mao County, Sichuan Province, China	BJFUZLC055	ON167907
*Cotinus szechuanensis* 444	Li County, Sichuan Province, China	BJFUZLC444	ON167908

**TABLE A2:** List of samples and their GenBank accessions for cp genomes and ITS sequences obtained from GenBank in the study.

Samples	GenBank accession number	
	Cp genomes	ITS sequence
*Anacardium occidentale*	KY835877	KF664192
*Boswellia sacra**	KT934315	AF445880
*Commiphora foliacea**	MH041484	JN882681
*Cotinus coggygria*	MT876478	–
*Mangifera indica*	MN711724	KJ833764
*Mangifera persiciforma*	MN917208	–
*Mangifera sylvatica*	MN786795	AB071689
*Rhus ovata*	NC049061	MT722588
*Rhus potaninii*	MT230556	MT227654
*Rhus punjabensis*	MT230555	–
*Rhus typhina*	MT083895	MT227675
*Pistacia chinensis*	MK738124	MH711767
*Pistacia vera*	MN551174	MH444724
*Pistacia weinmaniifolia*	MF630953	KF664191
*Toxicodendron succedaneum*	MT211614	KP093196
*Toxicodendron sylvestre*	MT211615	KP093197
*Toxicodendron vernicifluum*	MK550621	FJ945939

*Note*: Outgroup species were marked with asterisk.
